# Age-related somatic mutations in the cancer genome

**DOI:** 10.18632/oncotarget.5685

**Published:** 2015-09-17

**Authors:** Brandon Milholland, Adam Auton, Yousin Suh, Jan Vijg

**Affiliations:** ^1^ Albert Einstein College of Medicine, Bronx, NY, USA

**Keywords:** genomics, sequencing, aging, somatic mutation, bioinformatics

## Abstract

Aging is associated with an increased risk of cancer, possibly in part because of an age-related increase in mutations in normal tissues. Due to their extremely low abundance, somatic mutations in normal tissues frequently escape detection. Tumors, as clonal expansions of single cells, can provide information about the somatic mutations present in these cells prior to tumorigenesis.

Here, we used data from The Cancer Genome Atlas (TCGA), to systematically study the frequency and spectrum of somatic mutations in a total of 6,969 patients and 34 different tumor types as a function of the age of the patient. After using linear modeling to control for the age structure of different tumor types, we found that the number of identified somatic mutations increases exponentially with age. Using additional data from the literature, we found that accumulation of somatic mutations is associated with cell division rate, cancer risk and cigarette smoking, with the latter also associated with a distinct spectrum of mutations.

Our results confirm that aging is associated with the accumulation of somatic mutations, and strongly suggest that the level of genome instability of normal cells, modified by both endogenous and environmental factors, is the main risk factor for cancer.

## INTRODUCTION

Somatic mutations are generally accepted to cause cancer and have also been implicated as a cause of aging [1]. Transgenic reporter assays in mice and fruit flies have conclusively demonstrated that somatic mutations accumulate with age in a tissue-specific manner, with respect to both the rate of the age-related increase and the types of mutations found to accumulate [2–4]. But reporter genes may not always be representative of the genome overall, and with the emergence of next-generation sequencing it has become feasible to inexpensively characterize genome-wide, age-related mutation frequencies and spectra directly in different organs and tissues. As only one or few cells may contain the same somatic mutation, however, the detection of such mutations can be challenging even at very high depth. By contrast, somatic mutations are readily accessible in tumors, as these represent clonal expansions of the mutations in the original cell that gave rise to the tumor. Indeed, it is clear that the number of somatic mutations in tumors is significantly higher when the tumor was derived from an old patient as compared to a young one [5, 6]. Mathematical modeling strongly suggests that half or more of somatic mutations in tumors arise before initiation of the tumor, i.e., during development and aging. Hence, a considerable fraction of all mutations in a tumor may reflect the frequency and spectrum of somatic mutations in normal human cells as these accumulated with age [5]. Recent massive cancer-sequencing efforts, such as The Cancer Genome Atlas (TCGA), have made available a wealth of data on tumor-associated somatic mutations from many individuals and tissue types [7–9].

Thus far, a systematic analysis of mutation frequency and spectrum in human tumors as a function of the age of the patient has been lacking. Here, we fill this gap by studying a total of 6,969 patients with whole exome and/or whole genome sequencing data of mutations in 34 different types of tumors. The results show that the number of mutations in a tumor increases exponentially with age. Using linear modeling, we show that, despite differences between tissue types, this effect is robust and not an artifact of certain tumor types, which happen to have more mutations, appearing at later ages. Major differences in both mutation frequency and spectrum were observed between tumor types, with cell division rate and environmental exposure as the two main sources of variation. Our data underscore the finding that somatic mutation accumulation in normal cells, modified by both endogenous and environmental factors, is the main risk factor for cancer.

## RESULTS

### Somatic mutation frequency increases exponentially with patient age

**Figure 1:**
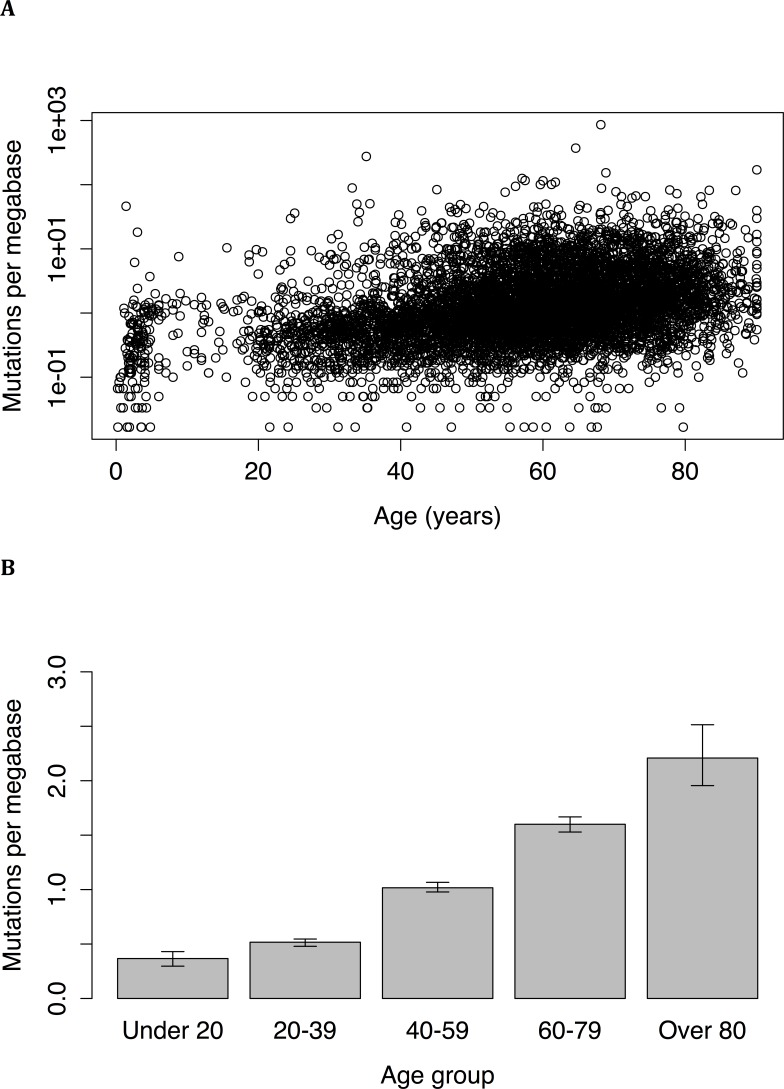
**(A)** Mutation frequency versus age in tumors of 6,969 individuals. The relationship between the two variables can be expressed as an exponential increase (*P* < 2.2*10^−16^, r = 0.36). **(B)** Frequency of somatic mutations in different age groups. Subjects over 80 had a mutation frequency more than 5 times higher than that of subjects under 20; the differences between all age groups are significant as measured by the Wilcoxon rank sum test.

Whole exome sequence data from a total of 6,969 individuals, with 34 different types of tumors, was examined. Across all samples and tumor types, the number of mutations was found to increase with age. Although a linear correlation to the untransformed data was statistically significant (*P* = 2.6*10^−10^, r = 0.076), a better fit (*P* < 2.2*10^−16^, r = 0.36) was obtained following log-transformation of mutation frequency (Figure 1A). Age was still significantly associated with mutation frequency even when tumors from juvenile patients (age less than 18) were excluded (*P* < 2.2*10^−16^, r = 0.33). The difference in mutation frequency between young and old individuals was very large: tumors from under 20 years old had a median mutation frequency of 0.37 mutations per megabase (95% CI = 0.30 to 0.43), while tumors from patients over 80 years old had a median mutation frequency of 2.21 mutations per megabase (95% CI = 1.96 to 2.51), representing a 6-fold increase over the course of a lifetime (Wilcoxon test: *P* < 2.2*10^−16^; Figure 1B). A robust regression also found a significant correlation (*P* < 2*10^−16^) between age and mutation frequency.

To jointly estimate the age-related increase in mutation frequency while accounting for cancer type, a linear model of log-transformed mutation frequency as a function of age and tumor type was created, such that $y_{i}=\beta x_{i}+\sum_{j=1}^{T}\gamma_{j}t_{i}+\varepsilon_{i}$, where *y_i_* represents the log-transformed mutation frequency in sample *i*, *x_i_* represents the sample age, *t_i_* represents a dummy variable indicating one of *T* tumor types, and *ɛ_i_* represents the residual for sample *i* This gave a better fit (r = 0.80) than any of the previous models; a model with an additional term for the interaction between tumor type and age did not produce a better fit and was not considered for further analysis. Results of the linear model are summarized in Supplementary Table 1. In this model, age was still found to be associated with mutation frequency (*P* < 2*10^−16^), accounting for a lifetime increase of 1.17 mutations per megabase between birth and age 80. Depending upon the tumor type, the estimated lifetime mutation accumulation varied from 0.084 in the case of rhabdoid tumors to 4.36 in the case of melanoma.

**Figure 2:**
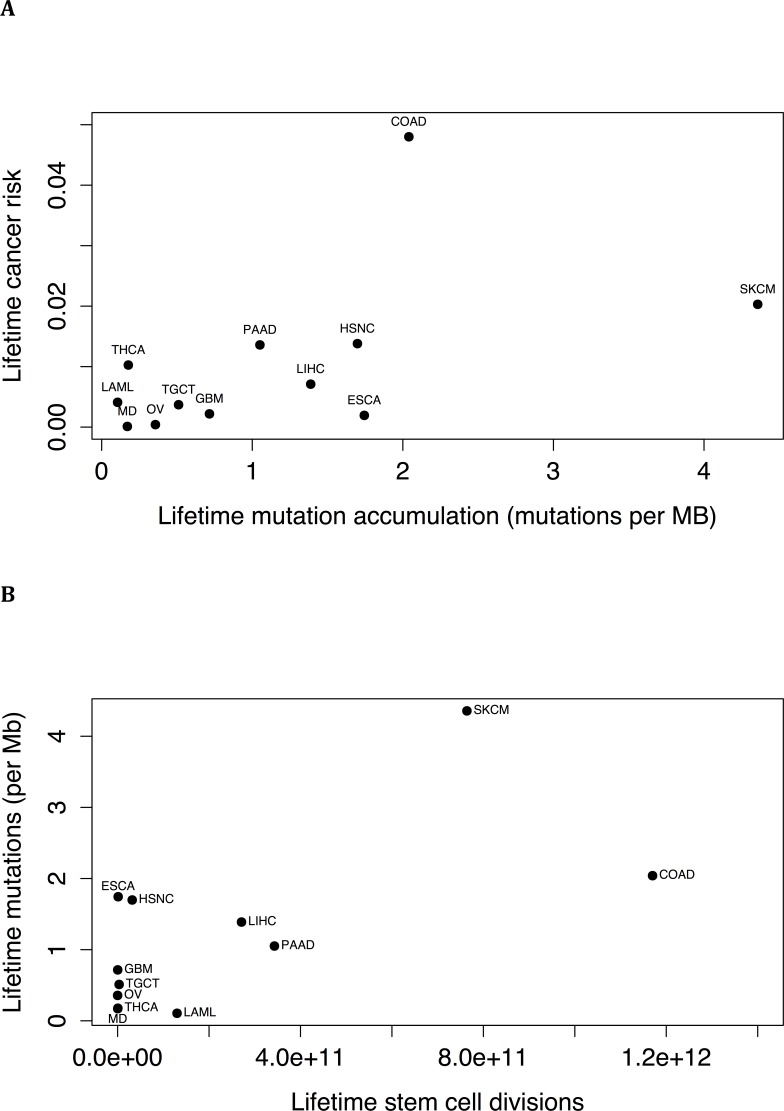
**(A)** Lifetime risk of cancer of a tissue type [10], as a function of the estimated lifetime mutation accumulation, i.e., the increase in mutation frequency calculated for the tissue type by the linear model between birth and age 80 (*P* = .079, r = 0.53). **(B)** Lifetime mutation accumulation, i.e. the increase in mutation frequency calculated for the tissue type by the linear model between birth and age 80, for different tissue types as a function of the estimated lifetime number of stem cell divisions (*P* = .019, r = 0.66). (Abbreviations: LAML=acute myeloid leukemia, COAD=colorectal adenocarcinoma, ESCA=esophageal squamous cell carcinoma, GBM=glioblastoma, HSNC=head and neck squamous cell carcinoma, LIHC=liver hepatocellular carcinoma, MD=medulloblastoma, SKCM=skin cutaneous melanoma, OV=ovarian, PAAD=pancreatic ductal adenocarcinoma, TGCT=testicular germ cell cancer, THCA=thyroid papillary/follicular carcinoma).

The cumulative number of stem cell divisions has been implicated as being a major risk factor for cancer [10]. We correlated the data from reference [10] with the results of our linear model. The association between lifetime mutation accumulation and lifetime cancer risk (Figure 2A) trended towards significance (*P* = .079, r = 0.53), and there was a significant correlation between lifetime mutation accumulation (*P* = .019, r = 0.66) and cumulative number of stem cell divisions (Figure 2B).

The spectrum of mutations did not remain constant throughout age. Linear models (summarized in Supplementary Table 2) as above, except with *y_i_* representing the proportion of a particular mutation in sample *i*, found an age-related increase in the proportion of C->T (*P* = .0042) and T->G mutations (*P* = .04) and an age-related decrease in the proportion of C->A (*P* = 7.28*10^−5^) mutations.

### Whole-exome data are representative for the whole genome

**Figure 3:**
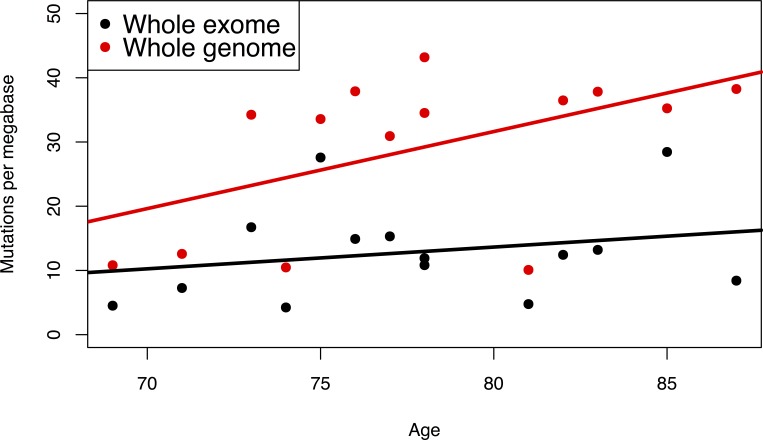
Mutation frequency as a function of age in 14 bladder tumors for which both whole exome and whole genome were available [11] Whole exome data: r = 0.50; whole genome data: r = 0.29.

To test if the exomic mutation frequency is representative of the mutation frequency in the overall genome, we compared mutation frequencies in a set of 14 bladder tumors for which both whole-exome and whole-genome mutation frequencies were available [11]. When considering the relationship between age and mutation frequency, the correlation was stronger with the genomic mutation frequency than with the exomic mutation frequency (r = 0.5 and r = 0.287, respectively; Figure 3). This suggests that the frequency of mutations in the exome is indicative of the frequency of mutations in the whole genome and that using the exomic mutation rate may even underestimate the strength of the relationship with age as opposed to using the genomic mutation rate. Therefore, an age-related increase in the whole-exome mutation frequency likely reflects a genome-wide increase in mutation frequency.

### Tissue-specific mutation rates and spectra

Previous studies of mutations in reporter genes in mice have found distinct frequencies and spectra of mutations in different tissue types [12]. Specifically, mice accumulated more mutations in the small intestine than in the heart, liver or spleen, which in turn accumulated more mutations than the brain [12]. Comparison of the tumor types found in our dataset yielded much the same results. In our data, the brain tumor types of glioblastoma multiforme, brain lower grade glioma and medulloblastoma had estimated lifetime mutation accumulations of 0.72, 0.56 and 0.17 mutations per megabase, all of which are below the median of 0.82 mutations per megabase. Rhabdoid tumors, which can also occur in the brain, had the lowest estimated lifetime mutation accumulation of any tumor type, 0.08. Liver hepatocellular carcinoma had an intermediate lifetime mutation accumulation of 1.39 mutations per megabase, less than one standard deviation (0.95) above the median. Finally, colon adenocarcinoma had an estimated lifetime mutation accumulation of 2.04 mutations per megabase.

Reporter gene studies in mice have also found an enrichment in all point mutation types other than C->G in small intestine compared to brain. The findings of our linear models relating tumor type and mutation spectra were in partial agreement with this result. Although we observed the enrichment for C->A and C->T mutations, with *γ_j_* for those mutations being higher in colorectal adenocarcinoma than the average for the brain-related tumor types, as well as a lack of enrichment of C->G mutations, with *γ_j_* being lower in colorectal adenocarcinoma, we did not observe an enrichment for T->A, T->C or T->G mutations; for those mutation types, *γ_j_* in colorectal adenocarcinoma was less than or equal to that in the brain-related tumor types. These differences between the mouse and human data may be species-specific, but could also be due to differences in cell type. Indeed, while the mouse reporter models analyzed all cells in a tissue, tumors are derived from specific cells, such as stem cells. Alternatively, these differences may be an artifact of the reporter gene system; not all mutations in the reporter gene would lead to a visible phenotype [13], so the spectrum of mutations found in the reporter gene would be biased towards those most likely to produce a phenotype.

To further examine the heterogeneity between tumor types, a separate exponential regression between mutation frequency and age was performed for each tumor type (summarized in Supplementary Table 3). At first, lifetime mutation accumulation, estimated by the difference between the frequency of mutations at age 80 and at birth, seemed to be poorly correlated (*P* = .46, r = −0.14) between the linear model and the separate regressions. However, this was mainly due to juvenile tumors having extremely high estimated amounts of estimated mutation accumulation and lung tumors having negative estimated mutation accumulation. Once both of these classes of tumors were removed, the correlation between the two estimates of lifetime mutation accumulation in the remaining 28 tumor types was highly significant (*P* = .00024, r=0.64).

The correlation coefficient varied between tumor types, from 0.4749 to −0.1771. The evidence was still overwhelmingly in favor of an age-related increase in mutation frequency, with the median correlation coefficient being 0.1993 and 29 of 34 tumor types having a positive correlation coefficient. The five tumor types with a negative correlation coefficient were: sarcoma, uveal melanoma, rectum adenocarcinoma, lung adenocarcinoma and lung squamous cell carcinoma. Sarcoma is primarily a juvenile cancer (median age: 6), so the lack of a positive correlation between age and mutation frequency is probably due to the low and narrow age range of patients. The only tumor types with negative correlation coefficients for which the *P* value was also significant were the two lung cancer types (*P* = .0099 and *P* = .019). We hypothesized that the effects of smoking may have concealed any age-related increase in mutation frequency and describe below our findings on the effects of smoking on mutation frequency.

**Figure 4:**
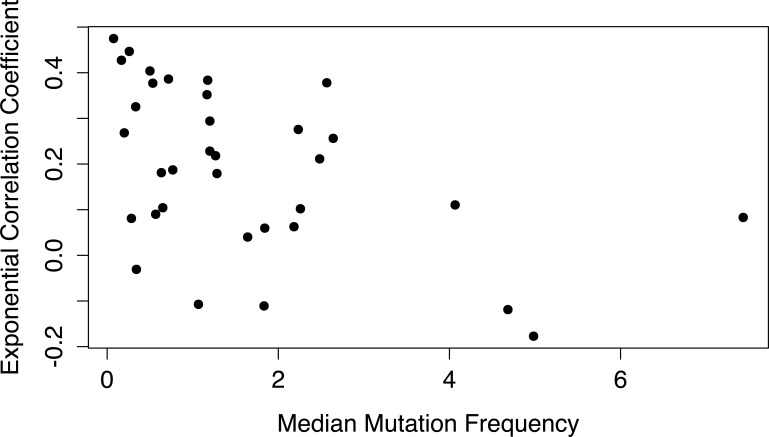
Correlation coefficient of mutation frequency increase with age as a function of median mutation frequency in the different tumors For each tumor type, the exponential correlation coefficient for the association between mutation frequency and age was plotted against its median mutation frequency. The correlation coefficients were inversely correlated with median mutation frequency (P = 0.0059, r = −0.46).

Even among adult tumor types without any known association with smoking, the correlation between age and mutation frequency was highly variable between tumor types. We hypothesized that tumor types with a weaker association between age and mutation frequency were prone to mutator phenotypes, in which tumors rapidly accumulate somatic mutations due to, e.g., mutations in DNA repair genes [14]. If this were the case, then we would expect that the tumor types with a lower correlation coefficient would have a higher median mutation frequency, which was indeed what we found: there was a significant negative correlation (*P* = .0059, r = −0.46) between median mutation frequency of a tumor type and its exponential correlation coefficient (Figure 4).

**Figure 5:**
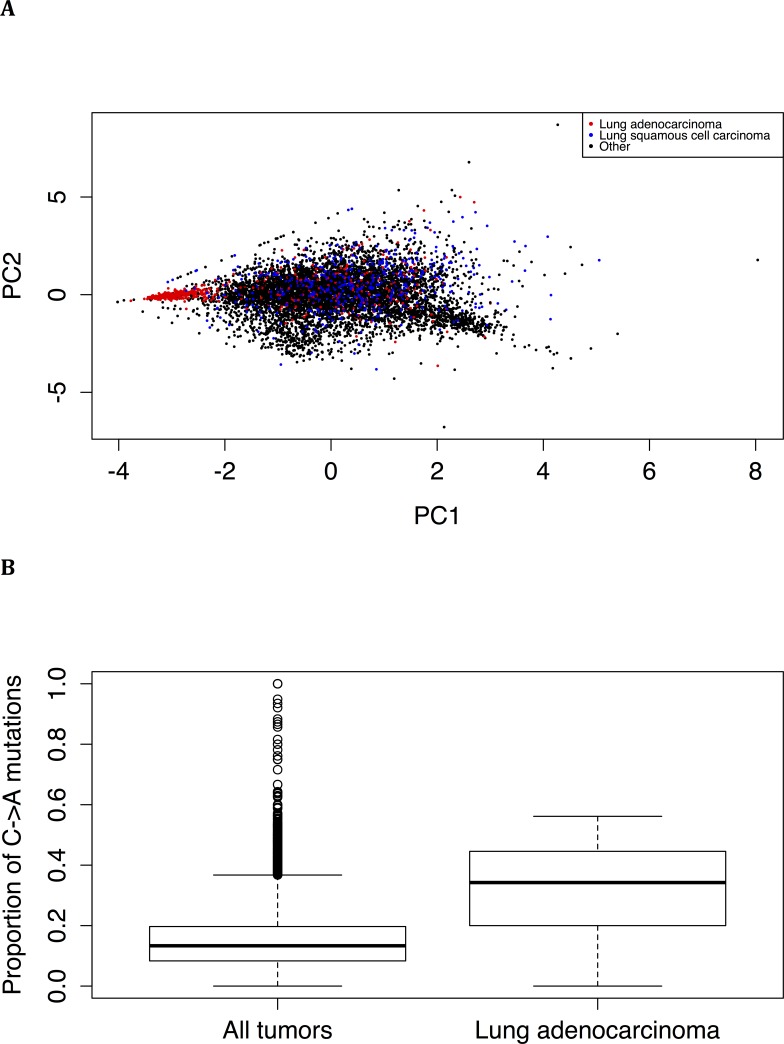
Distinct mutation spectrum in lung adenocarcinoma **(A)** Principal component analysis of the proportions of mutations reveals that lung adenocarcinoma tumors tend to have a spectrum of mutations not shared by other tumor types, including lung squamous cell carcinoma tumors. **(B)** Lung adenocarcinoma tumors have a larger proportion of C->A mutations than all tumor types combined (*P* < 2.2*10^−16^, Wilcoxon test).

Principal component analysis of the proportion of types of mutation found that lung adenocarcinoma tumors, but not lung squamous cell tumors, tend to have a distinct spectrum of mutations (Figure 5A). Lung adenocarcinoma tumors, compared to tumors overall, are significantly enriched (Wilcoxon test: *P* < 2.2*10^−16^) for C->A mutations (Figure 5B). This is consistent with the spectrum of mutations induced by tobacco smoke [15] and suggests that smoking has a strong effect on both the frequency and spectrum of somatic mutations (see also below).

### Effects of smoking on mutation frequency and spectrum

Information on the number of pack-years of smoking was available for 2,407 individuals. Another linear model (r = 0.82; summarized in Supplementary Table 4) was created to estimate the increase in mutation frequency due to smoking while controlling for both tumor type and age: $y_{i}=\beta x_{i}+\sum_{j=1}^{T}\gamma_{j}t_{i}+\partial p_{i}+\varepsilon_{i}$ (variables are the same as in the initial linear model, except *p_i_* represents the number of pack-years of smoking for sample *i*). Significant associations between mutation frequency and both age (*P* = .0012) and pack-years (*P* = 4.79*10-10) were found. Since *β* = 0.0061 and *∂* = 0.0038, a pack-year of smoking has the mutagenic equivalent of over 6 months of normal aging. Among smokers, the median number of pack-years was 39 and median age was 65. The number of additional mutations due to smoking 39 pack-years by age 65 is predicted to be between 0.1 and 0.63 per megabase, depending upon tissue type.

Smoking also altered the spectrum of somatic mutations. Linear modeling of the proportion of mutation types revealed that smoking significantly increases the proportion of C->A mutations (*P* = 3.41*10^−10^) and T->A mutations (*P* = 1.37*10^−10^), but significantly decreases the proportion of C->T mutations (*P* < 2*10^−16^).

## DISCUSSION

In this present study, we used the extensive amount of cancer genomic data available in TCGA to demonstrate a large and life-long increase in somatic mutation frequency across many tumor types. The relationship that we observed was best modeled as an exponential increase, which is consistent with a feedback loop in which somatic mutations lead to an overall decline of the functions of the cell, including genome maintenance, leading to even more somatic mutations. This large and prevalent increase strongly supports a possible role for the accumulation of somatic mutations in aging and cancer risk.

There was a correlation between number of cell divisions in a tissue and estimated lifetime mutation accumulation. Cell division, therefore, appears to be a major source of endogenous mutation. The association between lifetime mutation accumulation and lifetime cancer risk trended towards significance, consistent with somatic mutations playing a major role in cancer risk.

The strength of the correlation between age and mutation frequency varied greatly between tissue types. Although the preponderance of tumor types with a positive correlation strongly supported an age-related increase in somatic mutation frequency, for some tumor types the correlation coefficient was very low, or even negative. We found that for some tumor types, smoking acts to conceal the age-related increase in mutation frequencies; overall, the tumor types with higher median mutation frequencies have lower correlation coefficients, consistent with a mutator phenotype masking the age-related accumulation of somatic mutations. In this respect, it is possible that smoking affects mutation frequencies in tumor cells more readily than in normal cells (possibly because most mutations might be caused by replication errors), thereby promoting mutator phenotypes.

We observed an age-related spectrum of mutations, including an enrichment of C->T transitions, and a distinct spectrum of mutations associated with smoking, which included an enrichment of C->A transversions. The former is consistent with a widespread mutational signature previously found in other cancers and is thought to arise from spontaneous deamination of 5-methyl-cytosine, while the latter is consistent with a mutational signature found in lung cancers [15–17]. In addition to detecting the mutational signature of smoking, we were also able to quantify its relative contribution to the mutation frequency. Based on our linear model, one pack-year of smoking increases the somatic mutation frequency by the equivalent of over half a year of normal aging. Since the smokers in our data had consumed a median of 39 pack-years of cigarettes, the effect of smoking could be estimated to reduce lifespan by 23.7 years. Studies have shown that smoking reduces life expectancy by 11 years [18]; the overestimation of the lifespan reduction due to smoking is likely due to risk of mortality being affected by factors other than mutation frequency. Nonetheless, these findings suggest that somatic mutation frequency could be adopted as a way to predict the lifespan impact of other mutagens or lifestyle interventions.

There are two main limitations to this study: the possibility of post-tumorigenesis mutations affecting the results, and the possibility that the mutation frequencies in exomes are not representative of the genome as a whole. First of all, it is possible that in older patients, tumors have existed for a longer time and had a greater opportunity to accumulate mutations in cells within the tumor, which then expanded throughout the tumor. If this were the case, then the age-related increase in the frequency of tumor mutations would only reflect progression of the tumor and not the frequency of mutations in the cells prior to tumorigenesis. However, efforts at modeling the expansion of mutations within tumors have indicated that the majority of mutations found in tumors are present prior to tumorigenesis. They also indicate that the fraction of mutations present prior to tumor formation increases with age, so the expansion of mutations subsequent to tumorigenesis would tend to artificially decrease the correlation with age instead of increasing it [5].

Second, most of the mutation frequency values used for our study were obtained using whole-exome sequencing. Whole-genome mutation frequencies were available for a subset of tumors studied, and had a stronger correlation with age than the whole-exome mutation frequencies in the corresponding tumors. This is in keeping with observations that mutation frequencies tend to be lower in actively transcribed genes, possibly because of transcription-coupled repair. Therefore, the age-related increase in mutation frequency observed by whole-exome sequencing is more likely to be an underestimate than an overestimate of the age-related increase in whole-genome mutation frequency.

Our present findings constitute the most convincing evidence thus far of a widespread, age-related accumulation of somatic mutations in diverse human tissues. Decreased sequencing costs are likely to generate more whole-genome sequencing information, not only allowing confirmation of the accuracy of our results but also a more comprehensive analysis of patterns of mutations across the genome. Meanwhile, the observed accumulation of somatic mutations in a broad spectrum of human tissues does not provide evidence that mutations contribute to age-related diseases other than cancer. However, the magnitude of the observed mutation frequencies, i.e., varying from about 0.02 to almost 1,000 mutations per megabase, suggests that cell function can be affected directly without the need for clonal expansion and selection. Indeed, there has been an increasing interest in genome mosaicism, as this emerges during development and aging, and a possible causal role of somatic mutations in diseases other than cancer [1, 19]. To study this more effectively, new approaches, including single cell sequencing [23] have emerged for detecting both somatic mutations and their possible consequences for the transcriptome directly, without the limitations and potential artifacts present when relying on tumor [21]. These techniques may shed more light on a possible role of somatic mutations in aging and age-related diseases other than cancer.

## MATERIALS AND METHODS

### Source of mutation data and data processing

Clinical data and exomic tumor point mutation frequencies from 6,955 individuals were obtained from reference [8] and reference [9]. For purposes of comparing mutation frequencies, only single base pair substitutions were considered. Whole-genome and whole-exome mutation frequencies, along with clinical information, for an additional 14 individuals were taken from reference [11].

### Statistical analysis

Statistical analysis was performed using R [22]. Correlations were estimated using Pearson's correlation coefficient on the age of patients and untransformed or log-transformed number of mutations. A linear model was fitted to the proportion of a substitution type or the log-transformed frequency of mutations as a function of age and tumor type, using the functions built-in to R. Comparisons between age groups were performed using the Wilcoxon rank sum test and 95% confidence intervals were determined using the Boot library [23]. Robust correlation was performed using the Robust library [24].

## SUPPLEMENTARY MATERIAL TABLES


