# Supplementary material for: Age-related somatic mutations in the cancer genome
Source: Oncotarget. 2015 Sep 17;6(28):24627–35. doi: 10.18632/oncotarget.5685 (PMC4694783; doi:10.18632/oncotarget.5685)
Supplement: Supplementary file 1 [file oncotarget-06-24627-s001.pdf]

Age-related somatic mutations in the cancer genome

Supplementary Material

Supplementary Table 1: Results of a linear model relating the mutation frequency with age and tumor type.

Tumor Type	γ_j*	Estimated mutations per megabase at birth	Estimated mutations per megabase at 80	Lifetime mutation accumulation
Age (β)*	0.01	1.00	2.17	1.17
Adrenocortical carcinoma	-0.09	0.91	1.98	1.07
Bladder urothelial carcinoma	0.70	2.02	4.37	2.35
Breast invasive carcinoma	-0.90	0.41	0.88	0.47
Cervical squamous cell carcinoma and endocervical adenocarcinoma	0.60	1.82	3.94	2.12
Cholangiocarcinoma	-0.38	0.69	1.49	0.80
Colon adenocarcinoma	0.56	1.75	3.79	2.04
Esophageal carcinoma	0.40	1.49	3.24	1.74
Glioblastoma multiforme	-0.49	0.61	1.33	0.72
Head and neck squamous cell carcinoma	0.38	1.46	3.15	1.70
Kidney chromophobe	-1.03	0.36	0.77	0.42
Kidney renal clear cell carcinoma	-0.21	0.81	1.76	0.95
Kidney renal papillary cell carcinoma	-0.46	0.63	1.37	0.74
Acute myeloid leukemia	-2.40	0.09	0.20	0.11
Brain lower grade glioma	-0.73	0.48	1.04	0.56
Liver hepatocellular carcinoma	0.17	1.19	2.58	1.39
Lung adenocarcinoma	0.83	2.29	4.95	2.67
Lung squamous cell carcinoma	0.93	2.53	5.49	2.96
Medulloblastoma	-1.92	0.15	0.32	0.17
Neuroblastoma	-1.57	0.21	0.45	0.24
Ovarian serous cystoadenocarcinoma	-1.18	0.31	0.66	0.36
Pancreatic	-0.10	0.90	1.95	1.05

**adenocarcinoma**				
**Pheochromocytoma and paraganglioma**	-1.59	0.20	0.44	0.24
**Prostate adenocarcinoma**	-0.85	0.43	0.92	0.50
**Rhabdoid tumor**	-2.63	0.07	0.16	0.08
**Rectum adenocarcinoma**	0.14	1.15	2.49	1.34
**Sarcoma**	0.10	1.10	2.38	1.28
**Skin cutaneous melanoma**	1.32	3.74	8.09	4.36
**Stomach adenocarcinoma**	0.24	1.27	2.75	1.48
**Testicular germ cell tumors**	-0.83	0.44	0.95	0.51
**Thyroid carcinoma**	-1.89	0.15	0.33	0.18
**Thymoma**	-1.30	0.27	0.59	0.32
**Uterine corpus endometrial carcinoma**	0.23	1.26	2.72	1.47
**Uterine carcinosarcoma**	-0.35	0.71	1.53	0.82
**Uveal melanoma**	-1.68	0.19	0.40	0.22

* The values calculated for the linear model $y_i = \beta x_i + \sum_{j=1}^{T} \gamma_j t_i + \epsilon_i$, where y_i represents the log-transformed mutation frequency in sample i, x_i represents the sample age and ϵ_i represents the residual for sample i.

Supplementary Table 2: Results of six linear models relating the proportions of different substitution types with age and tumor type.

Tumor Type	γ (C->A)*	γ (C->G)*	γ (C->T)*	γ (T->A)*	γ (T->C)*	γ (T->G)*
Age (β)*	-3.32E-04	-1.33E-05†	3.38E-04	-2.53E-05†	-5.43E-05†	8.66E-05
Adrenocortical carcinoma	0.17	0.18	0.36	0.04	0.20	0.05
Bladder urothelial carcinoma	0.14	0.24	0.48	0.03	0.09	0.02
Breast invasive carcinoma	0.16	0.14	0.47	0.06	0.12	0.05
Cervical squamous cell carcinoma and endocervical adenocarcinoma	0.09	0.23	0.54	0.02	0.09	0.02
Cholangiocarcinoma	0.22	0.09	0.40	0.06	0.18	0.05
Colon adenocarcinoma	0.16	0.05	0.61	0.04	0.10	0.03
Esophageal carcinoma	0.18	0.12	0.43	0.05	0.14	0.07
Glioblastoma multiforme	0.12	0.08	0.60	0.05	0.13	0.03
Head and neck squamous cell carcinoma	0.17	0.16	0.47	0.06	0.12	0.03
Kidney chromophobe	0.15	0.06	0.45	0.05	0.26	0.04
Kidney renal clear cell carcinoma	0.20	0.11	0.35	0.11	0.17	0.06
Kidney renal papillary cell carcinoma	0.17	0.12	0.32	0.10	0.22	0.07
Acute myeloid leukemia	0.13	0.06	0.60	0.05	0.14	0.03
Brain lower grade glioma	0.11	0.09	0.53	0.04	0.20	0.04
Liver hepatocellular carcinoma	0.22	0.08	0.31	0.09	0.25	0.05
Lung adenocarcinoma	0.35	0.14	0.30	0.08	0.10	0.03
Lung squamous cell carcinoma	0.35	0.16	0.28	0.08	0.11	0.02
Medulloblastoma	0.15	0.08	0.59	0.04	0.12	0.02
Neuroblastoma	0.43	0.10	0.30	0.06	0.07	0.04
Ovarian serous cystoadenocarcinoma	0.19	0.17	0.38	0.08	0.13	0.05
Pancreatic adenocarcinoma	0.13	0.07	0.55	0.04	0.16	0.04
Pheochromocytoma and paraganglioma	0.12	0.10	0.46	0.06	0.23	0.04
Prostate	0.15	0.09	0.50	0.05	0.17	0.05

**adenocarcinoma**						
**Rhabdoid tumor**	0.11	0.09	0.63	3.01E-05†	0.04	0.13
**Rectum adenocarcinoma**	0.16	0.07	0.59	0.05	0.10	0.03
**Sarcoma**	0.21	0.10	0.42	0.06	0.17	0.04
**Skin cutaneous melanoma**	0.07	0.04	0.77	0.03	0.07	0.02
**Stomach adenocarcinoma**	0.15	0.08	0.49	0.05	0.14	0.09
**Testicular germ cell tumors**	0.25	0.11	0.34	0.04	0.20	0.07
**Thyroid carcinoma**	0.13	0.11	0.45	0.10	0.19	0.03
**Thymoma**	0.22	0.07	0.37	0.07	0.22	0.06
**Uterine corpus endometrial carcinoma**	0.20	0.08	0.51	0.04	0.13	0.03
**Uterine carcinosarcoma**	0.17	0.12	0.48	0.05	0.13	0.04
**Uveal melanoma**	0.11	0.10	0.47	0.08	0.18	0.07

*: The values calculated for the linear model $y_i = \beta x_i + \sum_{j=1}^{T} \gamma_j t_i + \epsilon_i$, where y_i represents the proportion of the relevant substitution type in sample i, x_i represents the sample age and ϵ_i represents the residual for sample i.

†: Not significant (p>0.05); for all others, the association was significant (p<0.05).

Supplementary Table 3: Results of separate exponential regressions on the data from each tumor type

Tumor Type	Median Mutation Frequency	*P* value	Correlation Coefficient*
**Acute myeloid leukemia**	0.20	1.68E-04	0.27
**Adrenocortical carcinoma**	1.28	0.09	0.18
**Bladder urothelial carcinoma**	4.07	0.03	0.11
**Brain lower grade glioma**	0.72	0.00E+00	0.39
**Breast invasive carcinoma**	0.65	3.73E-03	0.10
**Cervical squamous cell carcinoma and endocervical adenocarcinoma**	2.57	0.02	0.38
**Cholangiocarcinoma**	1.18	0.02	0.38
**Colon adenocarcinoma**	2.18	0.36	0.06
**Esophageal carcinoma**	2.64	6.42E-04	0.26
**Glioblastoma multiforme**	1.17	1.81E-09	0.35
**Head and neck squamous cell carcinoma**	2.48	1.70E-06	0.21
**Kidney chromophobe**	0.53	1.80E-03	0.38
**Kidney renal clear cell carcinoma**	1.27	1.35E-03	0.22
**Kidney renal papillary cell carcinoma**	1.20	1.82E-03	0.29
**Liver hepatocellular carcinoma**	2.26	0.15	0.10
**Lung adenocarcinoma**	4.68	0.01	-0.12
**Lung squamous cell carcinoma**	4.98	0.02	-0.18
**Medulloblastoma**	0.17	0.04	0.43
**Neuroblastoma**	0.28	0.48	0.08
**Ovarian serous cystoadenocarcinoma**	0.63	0.03	0.18
**Pancreatic adenocarcinoma**	1.84	0.49	0.06
**Pheochromocytoma and paraganglioma**	0.33	9.29E-06	0.33

**Prostate adenocarcinoma**	0.77	1.14E-04	0.19
**Rectum adenocarcinoma**	1.83	0.33	-0.11
**Rhabdoid tumor**	0.08	0.06	0.47
**Sarcoma**	1.07	0.39	-0.11
**Skin cutaneous melanoma**	7.43	0.12	0.08
**Stomach adenocarcinoma**	2.23	0.01	0.28
**Testicular germ cell tumors**	0.57	0.30	0.09
**Thymoma**	0.50	4.35E-06	0.40
**Thyroid carcinoma**	0.26	0.00E+00	0.45
**Uterine carcinosarcoma**	1.20	0.09	0.23
**Uterine corpus endometrial carcinoma**	1.64	0.58	0.04
**Uveal melanoma**	0.34	0.79	-0.03

*: Person's correlation coefficient of the correlation between patient age and log-transformed mutation frequency.

Supplementary Table 4: Results of a linear model containing parameters for age, tumor type and pack-years of smoking.

Tumor Type	γ_j*	Estimated mutations per megabase at birth	Estimated mutations per megabase after 65 years and no smoking	Estimated mutations per megabase after 65 years and 39 pack years	Additional mutations per megabase due to smoking
**Age (β)***	0.0061	1.00	1.49	NA	NA
**Packyears (∂)***	0.0038	1.00	1.28	1.72	0.44
**Bladder urothelial carcinoma**	0.80	2.24	3.32	3.86	0.53
**Cervical squamous cell carcinoma and endocervical adenocarcinoma**	0.63	1.87	2.79	3.23	0.44
**Esophageal carcinoma**	0.53	1.70	2.53	2.93	0.40
**Head and neck squamous cell carcinoma**	0.44	1.56	2.32	2.69	0.37
**Kidney chromophobe**	-0.83	0.44	0.65	0.75	0.10
**Kidney renal clear cell carcinoma**	-0.25	0.78	1.16	1.34	0.18
**Kidney renal papillary cell carcinoma**	-0.27	0.76	1.13	1.31	0.18
**Lung adenocarcinoma**	0.92	2.50	3.72	4.32	0.59
**Lung squamous cell carcinoma**	0.97	2.64	3.92	4.54	0.63
**Pancreatic adenocarcinoma**	0.22	1.25	1.85	2.15	0.30

*: Values calculated for the linear model $y_i = \beta x_i + \sum_{j=1}^{T} \gamma_j t_i + \partial p_i + \epsilon_i$, where y_i represents the log-transformed mutation frequency in sample i, x_i represents the sample age, p_i represents the number of pack-years of smoking for sample i, and ϵ_i represents the residual for sample i.
